# The Young-Feynman controlled double-slit electron interference experiment

**DOI:** 10.1038/s41598-019-43323-2

**Published:** 2019-07-18

**Authors:** Amir H. Tavabi, Chris B. Boothroyd, Emrah Yücelen, Stefano Frabboni, Gian Carlo Gazzadi, Rafal E. Dunin-Borkowski, Giulio Pozzi

**Affiliations:** 1 Ernst Ruska-Centre for Microscopy and Spectroscopy with Electrons and Peter Grünberg Institute, Forschungzentrum Jülich, 52425 Jülich, Germany; 2 School of Materials Science and Engineering, Nanyang Technological University, 50 Nanyang Avenue, Singapore, 639798 Singapore; 3 Thermo Fisher Scientific, Achtseweg Noord 5, 5600 KA Eindhoven, The Netherlands; 4 Department FIM, University of Modena and Reggio Emilia, via G. Campi 213/a, Modena, 41125 Italy; 5 CNR-Institute of Nanoscience-S3, via G. Campi 213/a, Modena, 41125 Italy; 6 Department of Physics and Astronomy, University of Bologna, viale B. Pichat 6/2, Bologna, 40127 Italy

**Keywords:** Quantum optics, Transmission electron microscopy

## Abstract

The key features of quantum mechanics are vividly illustrated by the Young-Feynman two-slit thought experiment, whose second part discusses the recording of an electron distribution with one of the two slits partially or totally closed by an aperture. Here, we realize the original Feynman proposal in a modern electron microscope equipped with a high brightness gun and two biprisms, with one of the biprisms used as a mask. By exciting the microscope lenses to conjugate the biprism plane with the slit plane, observations are carried out in the Fraunhofer plane with nearly ideal control of the covering of one of the slits. A second, new experiment is also presented, in which interference phenomena due to partial overlap of the slits are observed in the image plane. This condition is obtained by inserting the second biprism between the two slits and the first biprism and by biasing it in order to overlap their images.

## Introduction

Recent advances in electron optics, nanotechnology and specimen preparation have resulted in many studies on the experimental realization of the double-slit thought or gedanken experiment, which was described by Feynman as containing all of the mysteries of quantum mechanics^1,2^, using single free electrons.

The Young-Feynman experiment consists of three parts. The first part involves the observation of interference fringes in a double slit setup^3–6^ and their build-up using single electrons^7,8^. Two beam interference patterns can be observed using a Möllenstedt-Düker electron biprism^9,10^, which has proved to be the most versatile method for carrying out interferometry and holography experiments (see, *e*.*g*.^11–13^ for reviews). The build-up of two beam Fresnel interference fringes using single electrons was first demonstrated using an electron biprism as a wavefront beam splitter^14,15^. The second part of the Young-Feynman experiment involves a comparison of electron distributions recorded before and after one of the slits is closed^16^. Its analysis leads to the idea of the probability amplitude. It has been performed in a controlled manner by stopping one of the two beams in the Fraunhofer image of an electron biprism^17^ or in the Fresnel image of two slits^8^. The third part of the Young-Feynman experiment, which has subsequently been renamed the *which-way* (or *which-path*) experiment, aims at demonstrating that interference phenomena disappear when the setup is modified to obtain information about which slit the electron passes through. First experiments in this direction have been carried out by preparing nano-slits and depositing a layer of amorphous material using modern nanotechnology tools on one^18^ or both^19^ of them. Inelastic scattering in the material can be regarded as a dissipative process during the interaction, which is responsible for the localization mechanism^20,21^.

Here, we focus on the second part of the Young-Feynman experiment, which refers to the change in the interference pattern when one of the two slits is partially or totally obstructed in a controllable way. It is then possible to observe the transition of the diffraction pattern from the two- to the one-slit configuration, highlighting the wave-particle duality of the electrons. Although conceptually and mathematically simple, at least from the point of view of wave optical analysis^22,23^, its experimental realization is a very challenging task that requires advanced technology and instrumentation, as demonstrated by the partial success of former attempts^8,17^. After describing the drawbacks of former setups, we show how they can now be overcome by using a modern electron holography microscope that is equipped with two electron biprisms and a high brightness gun and how a new version of the experiment, in which the fringes are observed in the image instead of the Fraunhofer plane, can be realized.

## Analysis of Earlier Experiments

We first recall a few basic concepts in electron optics and microscopy. In general, the illumination system comprises an electron source followed by a system of condenser lenses, which demagnify the source, so that partial coherence does not blur the desired interference phenomena. As a result of the small de Broglie wavelength of high energy electrons, a further system of magnifying lenses is necessary so that interference fringe details can be resolved by an electron detector in the final recording plane. This plane can be optically conjugate to the observation plane OP, which can be the specimen plane if a gaussian image is desired, or the Fraunhofer diffraction plane (coincident with the back focal plane of the imaging lens for plane wave illumination) if a diffraction image is desired. The possibility of continuously varying the excitations of the electron lenses also allows planes in the Fresnel region between these two primary planes to be imaged.

**Figure 1:**
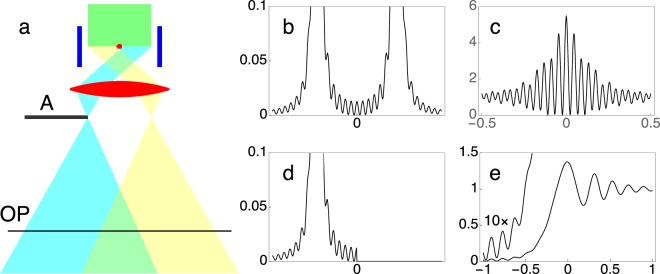
Controlled two beam experiment using an electron biprism. (**a**) Schematic diagram. (**b**) Simulated line profile across a Fraunhofer image of the biased electron biprism. (**c**) Corresponding Fresnel diffraction pattern in the observation plane OP. (**d**) Fraunhofer image of the biased electron biprism with an aperture A stopping the left beam. (**e**) Fresnel image of the complementary half-plane, with the fringes in the geometrical shadow region amplified by a factor of 10.

In the first experiment in which an electron biprism was used as an interferometry device^17^, a biprism was inserted in the normal specimen plane, where it could be biased in a specimen holder with contacts connected to an external voltage source. Two beam interference fringes could be observed in the observation plane OP, which was situated in the Fresnel region with respect to the biprism, as the standard gaussian image only showed its bare dark shadow. In order to stop one of the two beams passing to the left and right of the biprism, it was necessary to use an aperture A, which was inserted in a region where they were widely separated, corresponding approximately to the back focal plane of the imaging lens (*i*.*e*., the Fraunhofer plane), as shown in Fig. 1(a).

As a result of the features of the electron microscope that was used^17^, in which the aperture A was the selected area aperture, it was necessary to form the diffraction image of the biprism in this plane by working in the “low angle diffraction” mode^24^ by weakly exciting the standard objective lens (which then acted as a condenser lens) and using the following lens, the so-called diffraction lens, as an imaging lens. By changing the excitation of this lens, it was also possible to image a Fresnel plane, with the interference fringes in the overlapping region. This was not a real plane, as shown in Fig. 1 (for reasons of clarity), but a virtual plane in the actual experiment.

From the point of view of the interpretation and simulation of the results, it should be noted that not all of the experimental details are included explicitly in the simulations, which are usually referred to the object space with plane wave illumination. As the dimensions of the biprism and the slits are much larger than atomic dimensions, spherical aberration can be neglected and the standard theory of paraxial imaging, which has been described for both light optics^22,25^ and electron optics^23,26^, can be applied. When the process of image formation is described in the object space, propagation from the object to the Fraunhofer plane (*i*.*e*., the back focal plane of the imaging lens) can be described by a Fourier transform, while that from the back focal plane to the image plane can be described by an inverse Fourier transform. The effect of observing a Fresnel plane is accounted for simply by multiplying the spectrum by a quadratic phase factor^23,25^. This approach is also useful for numerical calculations, which make use of Fast Fourier Transforms. Here, Mathematica software^27^ was used to perform simulations.

Figure 1(a) illustrates the electron biprism setup that was used by Matteucci and Pozzi^17^. It involved the use of an aperture A to stop one of the two beams passing to the left and right of the biprism in a region where they were widely separated, corresponding approximately to the Fraunhofer plane behind the imaging lens. However, when the Fraunhofer image in this setup (Fig. 1(b)) is analyzed in detail, a weak system of diffraction fringes is found to be present in addition to the two bright spots that correspond to the two tilted half planes to the left and right of the biased biprism. These diffraction fringes are responsible for a modulation of the diffraction envelope of the corresponding two beam Fresnel interference image in the observation plane OP (Fig. 1(c)). As a result, even though aperture A in the Fraunhofer plane is able to completely block one of the spots, it fails to remove the faint fringes that originate from the Young diffracted waves at the edges of the biprism and the electrons are therefore not completely stopped (Fig. 1(d)). Both the simulation shown in Fig. 1(e) and the experiment^17^ show faint interference fringes (amplified by a factor of ten in Fig. 1(e)), primarily in the region of the geometrical shadow, where the intensity is expected to decrease continuously with distance for an opaque half plane^22^. The simulations start from the transmission function of the electron biprism^23^. Its Fourier Transform gives the Fraunhofer diffraction image shown in Fig. 1(b), while its Fresnel transform (which is obtained by multiplying the Fraunhofer diffraction image by the Fresnel factor) gives the image shown in Fig. 1(c). If an aperture is inserted in the Fraunhofer plane, then it cuts part of the Fraunhofer image (Fig. 1(d)) and its effect on the corresponding Fresnel image is shown in Fig. 1(e). A further drawback of this setup, which was used in previous experiments, was that experimental control over aperture A was not accurate enough to allow part of the diffracted spot to be intercepted, meaning that only open and closed states could be realized.

**Figure 2:**
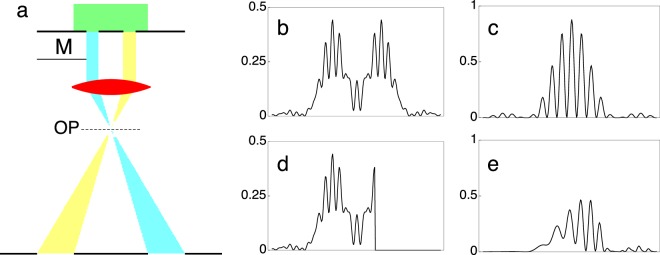
Controlled diffraction experiment with two slits and a mask M in the Fresnel region below the slits. (**a**) Scheme. (**b**) Fresnel image of the two slits in the plane of the mask M. (**c**) Corresponding Fraunhofer image. (**d**) Fresnel image of two slits with the mask M positioned exactly at the edge of the geometrical image of the right slit. (**e**) Corresponding Fraunhofer image.

Figure 2(a) shows the closest previous setup to the original Feynman proposal. This experiment was realized by Bach and co-workers^8^ on a dedicated electron optical bench and aimed to intercept electrons passing through one of two slits by using a movable mask M. However, as the mask could not be in the same plane as the slits but had to be placed some distance below them in the Fresnel region, it intercepted not the slits but their image in the mask plane (Fig. 2(b)). Observations were carried out by recording the Fraunhofer pattern of the slits (Fig. 2(c)). When the mask edge was exactly below the edge of the right slit (Fig. 2(d)), *i*.*e*., when all of the corresponding electrons should have been intercepted from the point of view of geometrical optics, a large fraction of them was still found to contribute to the Fraunhofer image, which displayed two beam interference fringes (Fig. 2(e)). These detrimental effects can be seen both in the experimental images of Bach and co-workers and in simulations reported in the Supplementary Information of their paper^8^. The simulations shown in Fig. 2 were carried out using their experimental parameters and confirm their results. The object transmission function is now that of two slits. The image is propagated to the aperture plane by multiplying the Fraunhofer image (Fig. 2(c)) by the Fresnel factor, resulting in Fig. 2(b). By taking into account the effect of the aperture (Fig. 2(d)), the original Fraunhofer image is modified, as shown in Fig. 2(e).

## The Ideal Controlled Beam Interference Experiment in the Fraunhofer Plane

A solution to the previous drawbacks and to the realization of a clear controlled beam interference experiment can be achieved by using an extra lens after the slits to form a magnified image of the slits instead of the Fraunhofer image. If an aperture is placed in such an intermediate image plane, which is conjugate to the object plane of the slits, then the ideal situation can be realized. The aperture acts as a virtual mask in the object plane, which can be used to cover one of the slits precisely, avoiding the detrimental effects that result from its placement in the Fresnel region.

**Figure 3:**
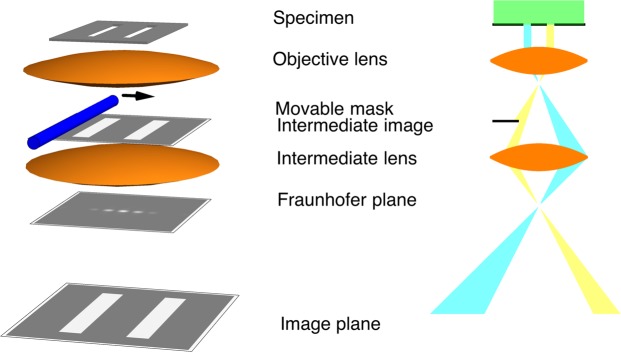
Sketch of the experimental setup (left) and of the corresponding ray path (right) for the ideal controlled two beam interference experiment in the Fraunhofer plane. An intermediate image of the two slits is formed by the objective lens in a plane in which a metallic wire, which is completely opaque to the electron beam, can be positioned with high accuracy to modify the electron transmittance through one of the two slits in a controlled way, acting in this way as a very sharp movable mask.. The position of the wire can be checked with the microscope in image mode. By switching the microscope to diffraction mode, it is possible to record the intensity in the Fraunhofer plane, thereby showing the fringe pattern of the Young-Feynman two slit experiment as a function of wire position, *i*.*e*., of the electron path localization.

A more detailed set up is shown in Fig. 3, which shows that the image of the two-slit specimen is focused by the objective lens in the intermediate image plane, where a biprism wire can be used as a very sharp mask. In the back focal plane of the intermediate lens, a Fraunhofer image is formed, followed, along the optical axis, by a gaussian image in the image plane. Both of these planes can be imaged by suitably exciting the remaining imaging lenses of the microscope, which are not shown in Fig. 3. The electron optical requirements that are necessary for realizing this experiment can now be satisfied, as described below.

**Figure 4:**
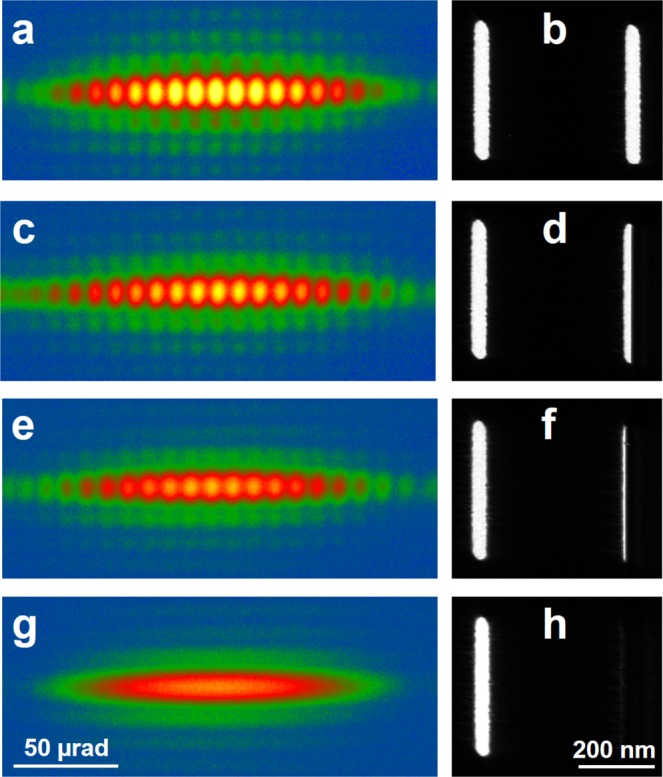
Two slit controlled electron beam experiment, showing the use of a mask placed in the conjugate plane of the slits to progressively cover the right-hand slit (right) and its effect on the corresponding Fraunhofer diffraction image (left).

Figure 4(a,c,e,g) shows experimental Fraunhofer images, which were recorded using a camera at the end of a post-column Gatan imaging filter (GIF) in Lorentz mode, in order to have a sufficiently long camera length to image the interference fringes within the central maximum of the diffraction envelope. It is interesting that the weak single slit diffraction fringes in the perpendicular (vertical) direction are nearly square, as the lengths of the slits are nearly equal to their separation (*e*.*g*.^6^). Focused images of the slits, which are shown in Fig. 4(b,d,f,h), were recorded both before and after recording the diffraction images, in order to detect any drift of the biprism. In Fig. 4(b), the right slit is completely open and a standard two beam interference image is obtained. In Fig. 4(d), the biprism has been moved in such a way that the right slit is approximately half open, whereas in Fig. 4(f) only a small fraction of the electron beam is allowed to pass through the right slit. In Fig. 4(h), the right slit is completely blocked, the interference fringes have disappeared completely and only the diffraction image of the open left slit remains.

**Figure 5:**
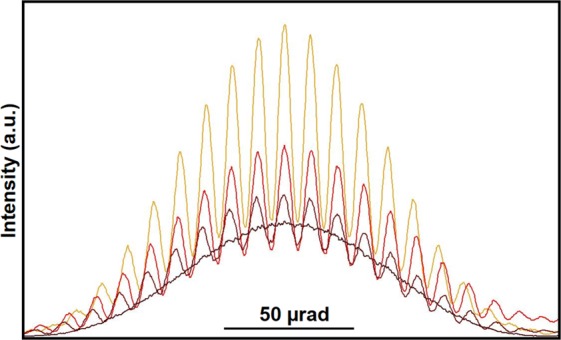
Line scans measured across the central maxima in the experimental Fraunhofer patterns shown in Fig. 4.

**Figure 6:**
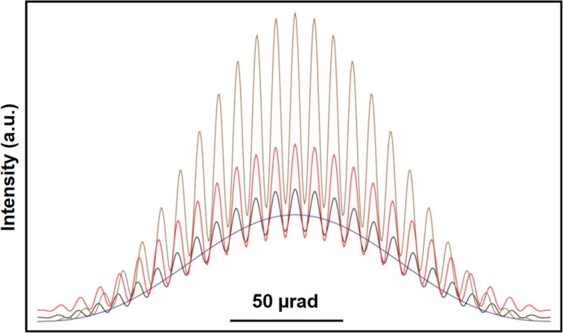
Simulated line scans of the Fraunhofer patterns generated assuming incoherent plane wave illumination with a gaussian distribution over a semi-angle of 2.8 10^−6^ rad.

The changes in the contrast and shape of the fringes in Fig. 4 can be examined more quantitatively by taking line scans across the central maximum of the diffraction envelope, as shown in Fig. 5. The decreasing contrast and intensity of the curves corresponds to the decreasing width of the covered slit. The line scans show that the fringe contrast is approximately 0.5 when the slits are both open, presumably because the electron beam illumination was not perfectly coherent. The observed contrast reduction can be simulated by introducing incoherent plane wave illumination with a gaussian distribution over a semi-angle of 2.8 10^−6^ rad. Figure 6 shows the result of convoluting the intensities of perfectly coherent images for two one-dimensional slits (with widths of 36 nm and a separation of 496 nm) with this angular distribution for partial coverages of the right slit of 0, 0.5, 0.8 and 1. The agreement with the experimental results is satisfying. Further details are reported in the Supplementary Information.

## A Controlled Beam Interference Experiment in the Image Plane

**Figure 7:**
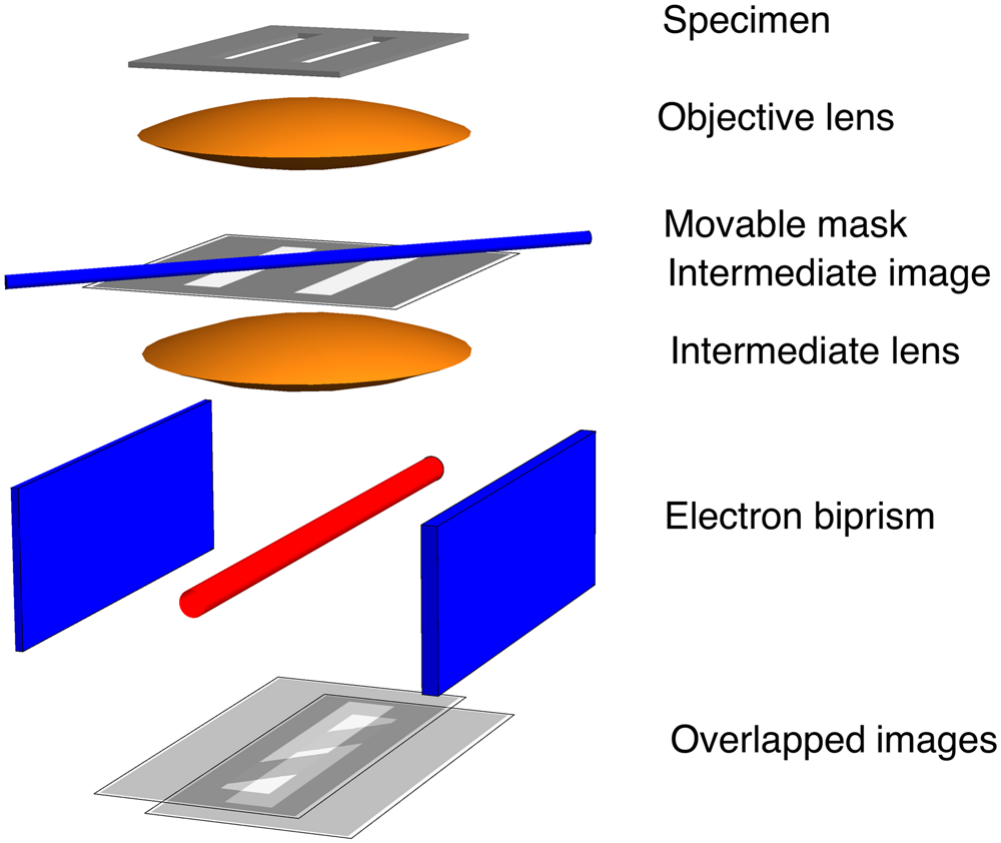
Sketch of the experimental setup for the ideal controlled two beam interference experiment in the image plane. Just as in Fig. 3, an intermediate image of the two slits is formed by the objective lens in a plane in which a metallic wire (the unbiased first biprism wire, shown in blue), which is opaque to the electron beam, can be positioned with high accuracy and acts as a very sharp movable mask. In this case, it is possible to locally modify the electron transmittance through both slits. A biased biprism (shown in red), which is positioned in a plane above the second intermediate image plane and held at an applied voltage with respect to ground, acts as a wavefront division interferometric device. The resulting tilted wavefronts are overlapped in the observation plane, which is conjugate to the detector plane, by the remaining lenses of the microscope. Interference fringes are observed, except in the shadow of the mask, where electron path localization is achieved by absorption from the first wire.

In the previous section, the electron microscope was essentially used as an electron optical bench, in order to bring the mask (biprism) plane conjugate to the slit plane and to capture the Fraunhofer diffraction image of the slits on the detector. Another intriguing experiment can be realized by recording interference fringes in the plane of the slits (*i*.*e*., the object plane) instead of in the Fraunhofer plane, as shown in Fig. 7. As the microscope used in the present study is equipped with two electron biprisms^28,29^, the first of which serves as a mask, the second biprism can be used in the same way as in a standard off-axis electron holography setup^11^, in order to overlap wavefunctions passing on its left and right side in the image plane (conjugate to the object plane). We believe that the paradoxical question of which slit the electron is passing through is even more emphasized by this setup, where the two slits are imaged together.

**Figure 8:**
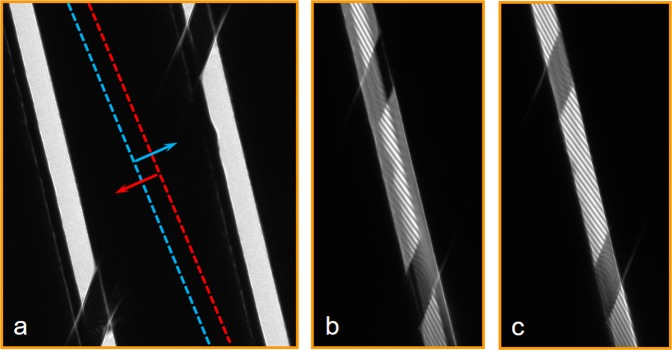
(**a**) Controlled electron beam interference experiments in image space recorded using two electron biprisms. The lower biprism, which is located in the dark region between the slits and positioned some distance above the slit image plane, is marked by dashed lines that indicate its edges. The arrows show the displacement of the two halves of the image when a bias is applied to the lower biprism. The upper biprism crosses the slits at an angle and is in a conjugate image plane to them. Partial (**b**) and total (**c**) overlap of the two slits is achieved when the bias applied to the lower biprism is increased, resulting in the formation of two beam interference fringes.

We carried out experiments at 300 kV (corresponding to a de Broglie electron wavelength of 1.97 pm) using slits of the same width and separation as before, but with a length of 2 *μ*m. The lower biprism was inserted between the slits, so that it was in the opaque region between them and could not be seen. Figure 8(a) shows the two edges of the lower biprism marked by dashed lines. As it is positioned above the slit image plane, when a bias is applied to it the two half-planes on its left and right sides shift in perpendicular directions, as shown by arrows. When a suitable potential is applied to the lower biprism, the two slits are brought to a partial (Fig. 8(b)) or nearly total (Fig. 8(c)) overlap. In the overlap region, interference fringes appear. Partial transparency of the upper biprism at 300 kV results in the presence of interference fringes in the regions that are shadowed by the upper biprism (where one beam is left).

Unfortunately, as the lower biprism is necessarily located in the Fresnel region below the two slits and the upper biprism, diffraction effects due to its sharp edges cannot be avoided. Moreover, selective filtering of the spatial frequencies by the lower biprism is responsible for the presence of ghost images of the edges of the upper biprism^11,30^, which can be seen more clearly in a dynamic way in the form of a movie in the Supplementary Information. Nevertheless, it is possible to identify single image areas where fringes from two slit interference are present and areas with no (or only faint) interference fringes where the slits are blocked by the upper biprism.

## Conclusions

We have shown how the second part of the Young-Feynman experiment, which leads to the physical concept of the probability amplitude, can be realized by using a modern electron microscope as a versatile electron optical bench and nanotechnology preparation methods to fabricate slits at the submicron level. Two versions of the experiment have been presented, one of which corresponds closely to the original proposal and is free from artefacts that plagued previous experiments, while the other is realized by using two electron biprisms to overlap the slits in the image plane.

## Methods

The experiments that are reported here were carried out in an FEI Titan 60–300 transmission electron microscope equipped with a high brightness electron gun, a Lorentz lens, a Gatan imaging filter (GIF), a 2048 × 2048 pixel charge-coupled device camera and two rotatable electron biprisms after the specimen plane. The microscope was operated primarily at an accelerating voltage of 60 kV (corresponding to a de Broglie electron wavelength of 4.87 pm) because the biprism wire was not completely opaque to 300 kV electrons. The lens excitations were chosen so that the upper biprism was in the first conjugate intermediate image plane. It could then be carefully aligned and displaced to obscure one of the slits either partially or totally, thanks to its very sharp edge when compared with the roughness of standard apertures.

The slits were designed so that they could be covered completely by the shadow of the upper electron biprism. They were fabricated using focused ion beam milling in a dual beam workstation (FEI Strata DB 235 M) on a commercial silicon nitride membrane window with a 200-*μ*m-thick Si frame and a 100 *μ*m × 100 *μ*m square window that comprised a bilayer of 200 nm of silicon nitride and a further 100-nm-thick Au film. In order to open the slits, a 9 pA Ga ion beam with a nominal spot size of 10 nm was scanned over two 30 nm × 480 nm boxes that were spaced 500 nm apart for 4 s for each box. As a result of the dimensions of the slits and the angular deflections that are involved, the lenses can be considered to be ideal and their geometrical and chromatic aberrations can be considered to be negligible.

### Note added during review

In the final stage of the review process, we became aware of a recently published paper by Harada *et al*.^31^ on interference experiments with asymmetric double slits, which were aimed at categorisation of the electrons. As their experiments were carried out using a 1.2 MV field emission electron microscope, the biprisms that were used as slits were not completely opaque to the electrons, as required in the experiments reported in the present work, in which the required opacity was achieved only at a very low accelerating potential of 60 kV.

## Supplementary information

Supplementary information

Deatest 1

## References

[1] Feynman RP, Leighton RB, Sands ML (1963). The Feynman lectures on physics.

[2] Crease RP (2003). The prism and the pendulum: the ten most beautiful experiments in science.

[3] Jönsson C (1961). Elektroneninterferenzen an mehreren künstlich hergestellten Feinspalten. Zeitschrift für Physik.

[4] Jönsson C (1974). Electron diffraction at multiple slits. Am. J. Phys..

[5] Frabboni S, Gazzadi GC, Pozzi G (2007). Young’s double-slit interference experiment with electrons. Am. J. Phys..

[6] Frabboni S, Frigeri C, Gazzadi GC, Pozzi G (2011). Two and three slit electron interference and diffraction experiments. Am. J. Phys..

[7] Frabboni S (2012). The Young-Feynman two-slits experiment with single electrons: Build-up of the interference pattern and arrival-time distribution using a fast-readout pixel detector. Ultramicroscopy.

[8] Bach R, Pope D, Liou S-H, Batelaan H (2013). Controlled double-slit electron diffraction. New J. Phys..

[9] Möllenstedt, G., & H. Düker. Fresnelscher Interferenzversuch mit einem Biprisma für Elektronenwellen. *Naturwissenschaften* **42**(2), 41–41(1955).

[10] Möllenstedt G, Düker H (1956). Beobachtungen und Messungen an Biprisma-Interferenzen mit Elektronenwellen. Zeitschrift für Physik.

[11] Missiroli GF, Pozzi G, Valdrè U (1981). Electron interferometry and interference electron microscopy. J. Phys. E..

[12] Hasselbach Franz (2009). Progress in electron- and ion-interferometry. Reports on Progress in Physics.

[13] Pozzi G, Beleggia M, Kasama T, Dunin-Borkowski RE (2014). Interferometric methods for mapping static electric and magnetic fields. Comptes Rendus Physique.

[14] Merli PG, Missiroli GF, Pozzi G (1976). On the statistical aspect of electron interference phenomena. Am. J. Phys..

[15] Tonomura A, Endo J, Matsuda T, Kawasaki T, Ezawa H (1989). Demonstration of single-electron buildup of an interference pattern. Am. J. Phys..

[16] Frabboni S, Gazzadi GC, Pozzi G (2008). Nanofabrication and the realization of Feynman’s two-slit experiment. Appl. Phys. Lett..

[17] Matteucci G, Pozzi G (1978). Two further experiments on electron interference. Am. J. Phys..

[18] Frabboni, S., Gazzadi, G. C. & Pozzi, G. Ion and electron beam nanofabrication of the which-way double-slit experiment in a transmission electron microscope. *Applied Physics Letters* **97**(26), 263101 (2010).

[19] Frabboni S, Gazzadi GC, Grillo V, Pozzi G (2015). Elastic and inelastic electrons in the double-slit experiment: A variant of Feynman’s which-way set-up. Ultramicroscopy.

[20] Egerton RF (2007). Limits to the spatial, energy and momentum resolution of electron energy-loss spectroscopy. Ultramicroscopy.

[21] Egerton R F (2008). Electron energy-loss spectroscopy in the TEM. Reports on Progress in Physics.

[22] Born M, Wolf E (1969). Principles of optics: electromagnetic theory of propagation, interference and diffraction of light.

[23] Pozzi, G. Particles and waves in electron optics and microscopy. vol. 194 of *Advances in imaging and electron physics*. (Academic Press, New York, NY, 2016).

[24] Ferrier, R. P. Small angle electron diffraction in the electron microscope. In Barer, R. & Cosslett, V. E. (eds) *Advances in Optical and Electron Microscopy*, vol. 3, 155–217 (Academic Press, New York, 1969).

[25] Goodman JW (1996). Introduction to Fourier optics.

[26] Glaser W (1952). Grundlagen der Elektronenoptik.

[27] Wolfram S (1999). The Mathematica book.

[28] Harada K, Tonomura A, Togawa Y, Akashi T, Matsuda T (2004). Double-biprism electron interferometry. Appl. Phys. Lett..

[29] Harada K, Akashi T, Togawa Y, Matsuda T, Tonomura A (2005). Variable interference azimuth angle in double-biprism electron interferometry. Jpn. J. Appl. Phys..

[30] Faget J (1961). Interférences des ondes électroniques: application à une méthode de microscopie électronique interférentielle. Revue d’Optique.

[31] Harada K (2018). Interference experiment with asymmetric double slit by using 1.2-mv field emission transmission electron microscope. Sci. Rep..

