# Supplementary material for: The Young-Feynman controlled double-slit electron interference experiment
Source: Sci Rep. 2019 Jul 18;9:10458. doi: 10.1038/s41598-019-43323-2 (PMC6639407; doi:10.1038/s41598-019-43323-2)
Supplement: Supplementary file 1 — Supplementary information [file 41598_2019_43323_MOESM1_ESM.pdf]

The Young-Feynman controlled double-slit electron interference experiment. Supplementary Information.

Amir H. Tavabi[1,*], Chris B. Boothroyd[1,2], Emrah Yücelen[3], Stefano Frabboni[4,5], Gian Carlo Gazzadi [5], Rafal E. Dunin-Borkowski[1], and Giulio Pozzi[1,6]

[1]Ernst Ruska-Centre for Microscopy and Spectroscopy with Electrons and Peter Grünberg Institute, Forschungzentrum Jülich, 52425 Jülich, Germany
[*]a.tavabi@fz-juelich.de
[2]School of Materials Science and Engineering, Nanyang Technological University, 50 Nanyang Avenue, Singapore 639798
[3]FEI Company, Achtseweg Noord 5, 5600 KA, Eindhoven, The Netherlands
[4]Department FIM, University of Modena and Reggio Emilia, via G. Campi 213/a, Modena, 41125, Italy
[5]CNR-Institute of Nanoscience-S3, via G. Campi 213/a, Modena, 41125, Italy
[6]Department of Physics and Astronomy, University of Bologna, , viale B. Pichat 6/2, Bologna, 40127, Italy

ABSTRACT

Additional information is provided about the experiments that are described in the main paper and their theoretical interpretation and simulation.

S1 The ideal controlled beam interference experiment in the Fraunhofer plane

Figure S1 shows a scanning electron microscopy (SEM) image of the slits used in the experiment, recorded in a dual beam workstation (FEI Strata DB 235 M).

If we approximate the slits by rectangles with the dimensions indicated in the figure, then the wavefunction that propagates after the slits is separated into variables perpendicular and parallel to the slits. Apart form a scaling factor in intensity, we can then use one-dimensional calculations[1]. Although the wavefunction can be expressed in analytical form using Fresnel functions[1], it is better to use numerical simulations from the beginning, in order to use Fast Fourier Transforms to take into account the effect of partial spatial coherence by means of a convolution of the image intensity with the source function. The effect of partial temporal coherence, which linked to the energy spread of the electron source, is considered to be negligible here.

Slits with the dimension reported in Fig. S1 were centered in a field of view of 8 μm, corresponding to 4096 pixels. Calculations performed with larger numbers of pixels gave indistinguishable results. The accelerating potential of the electrons was 60 kV, with a corresponding de Broglie wavelength of 4.87 pm. Figure S2 shows the effect on the two beam interference fringes in the Fraunhofer plane of using a gaussian angular distribution $\exp[-\alpha^2/\alpha_{ill}^2]$ for various values of the illumination angle α_{ill}. The abscissae are labelled in pixels, each of which corresponds to an angular width of 0.608 μrad. The ordinates give the intensities, relative to unit amplitude and intensity in the slits in the object plane.

A satisfying value for the illumination when both slits are open is $\alpha_{ill} = 2.8$ μrad. Accordingly, Fig. S3 reports the effect of this illumination angle on the two beam Fraunhofer fringes when one of the slits is partially covered. The left column shows simulations for perfect coherence, while the right column shown simulations for partial coherence, which together form Fig. 5 in the paper.

S2 A controlled beam interference experiment in the image plane

In this experiment, we placed the biprism conjugate to the specimen, in order to mask out part of the slits[2] to cancel the interference fringes when the bright area of one slit was overlapped onto the dark area of the other slit. A movie was recorded when the potential of the second biprism was varied to partially or totally overlap the slits.

References

1. Pozzi, G. Particles and waves in electron optics and microscopy. vol. 194 of *Advances in imaging and electron physics* (Academic Press, New York, NY, 2016).
2. Tanigaki, T. *et al.* Split-illumination electron holography for improved evaluation of electrostatic potential associated with electrophotography. *Applied Physics Letters* **104**, 131601 (2014).

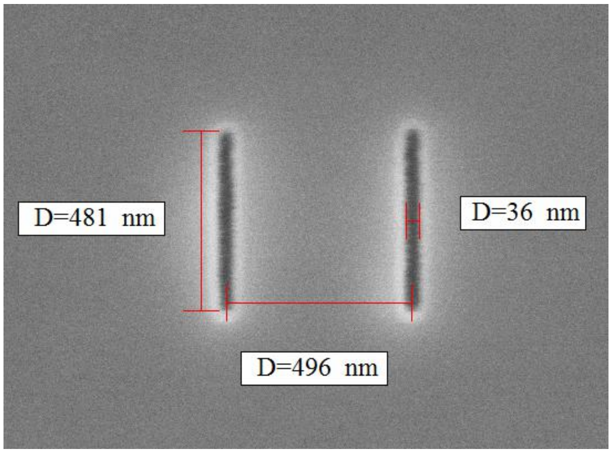

Figure S1. SEM image of the specimen.

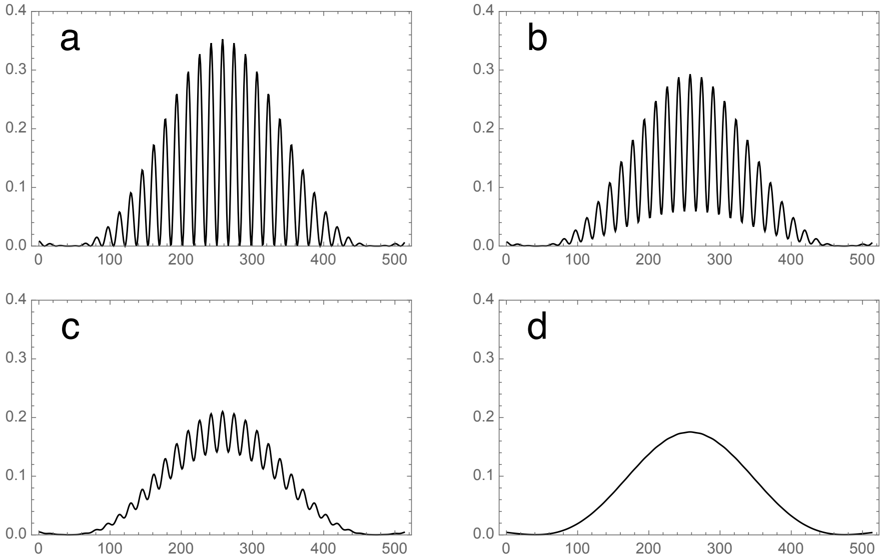

Figure S2. Effect of partial spatial coherence on the two beam interference images. Illumination angle: a) perfect coherence; b) 2 μrad; c) 4 μrad; d) 8 μrad.

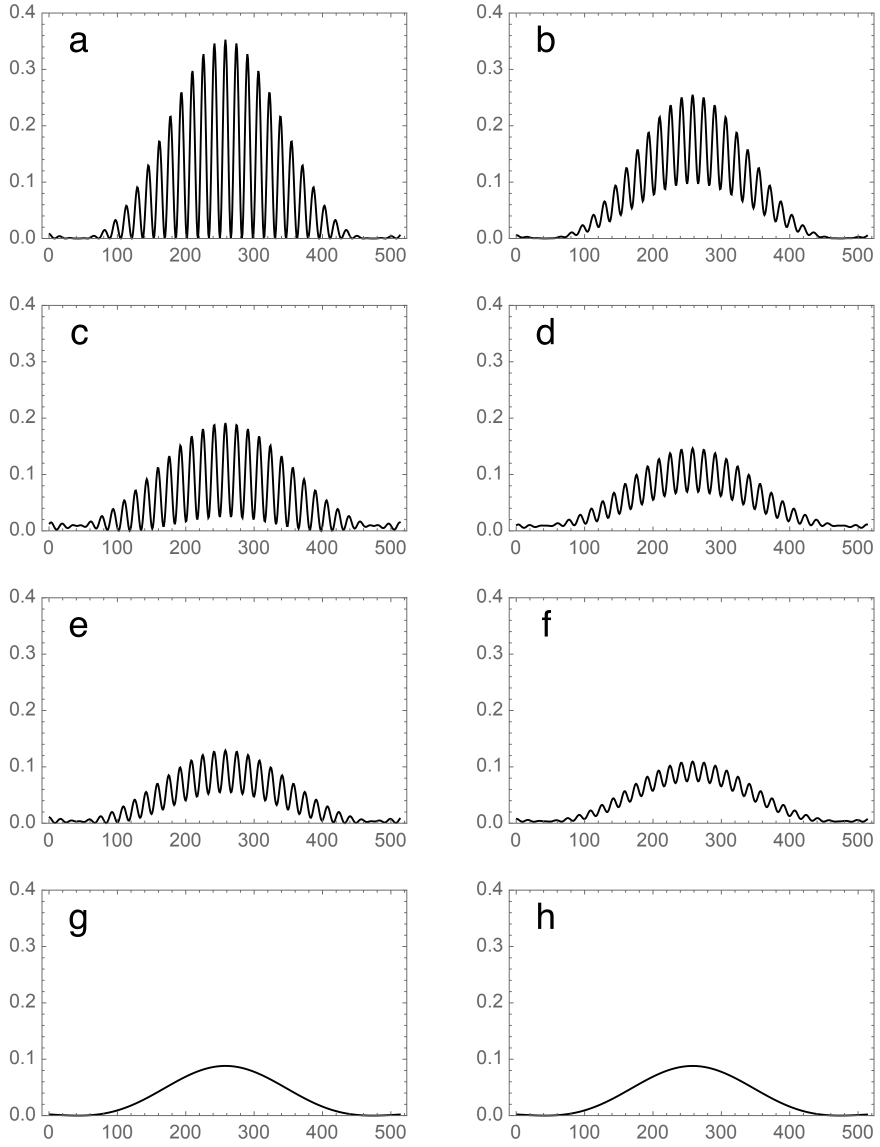

Figure S3. Effect of partial spatial coherence with an illumination angle of 2.8 μrad on two beam interference images with partial covering of one of the two apertures. The left column corresponds to perfect coherence. The right column is for partial coherence. The coverings are a) and b) 0; c) and d) 0.5; e) and f) 0.8; g) and h) 1.
